# A review of different types of volunteer programs for older adults with and without cognitive impairment

**DOI:** 10.3389/fpubh.2023.1164952

**Published:** 2023-06-15

**Authors:** Xianghe Zhu, Zhiwei Dong, Yili Wu, Dong-Wu Xu

**Affiliations:** ^1^Department of Psychology, School of Mental Health, Wenzhou Medical University, Wenzhou, Zhejiang, China; ^2^Oujiang Laboratory (Zhejiang Lab for Regenerative Medicine, Vision and Brain Health), Wenzhou, China; ^3^Key Laboratory of Alzheimer's Disease of Zhejiang Province, Institute of Aging, Wenzhou Medical University, Wenzhou, Zhejiang, China; ^4^Zhejiang Provincial Clinical Research Center for Mental Disorders, The Affiliated Kangning Hospital, Wenzhou Medical University, Wenzhou, China; ^5^College of Management, Weifang Medical University, Weifang, China

**Keywords:** volunteering, older volunteers, cognitive impairment, intervention, activity

## Abstract

Theoretical models and empirical evidence suggest an association between volunteering and health outcomes in older adults. However, less is known about existing programs that involve older adults engaging in formal volunteering, especially programs for older volunteers with cognitive impairment. In this review, we summarized and evaluated different types of volunteering-based programs involving older volunteers with and without cognitive impairment. After a non-systematic literature search, we presented eight example volunteer programs. Older volunteers participate in the programs in person or remotely. In five of the programs, older volunteers without cognitive impairment participate in intergenerational engagement, support and referral, home visiting, and dementia care services. The other three programs specifically recruit older volunteers with cognitive impairment and provide intergenerational engagement and individualized volunteer activities. Both strengths and challenges identified in the programs were discussed. Different types of volunteering-based programs are available for engaging older volunteers. For volunteers to remain active during the pandemic or for volunteers who live with cognitive impairment, remote programs can be a valuable alternative. Program effects on older volunteers need to be tested in more rigorously designed studies.

## Introduction

Volunteering is defined as helping behavior chosen by the individual and offered to strangers not for monetary gains, and formal volunteering, in particular, is often planned and ongoing within an organizational setting (1, 2). Theoretical models (3) and empirical evidence (4) suggest an association between volunteering and healthier outcomes in older adults. However, compared to other lifestyle interventions for healthy aging [e.g., physical activity (5)], less is known about existing volunteering-based programs that target older volunteers, especially regarding variations in their goals, designs, and effects. Emerging evidence also suggests that volunteering can benefit people with cognitive impairment (6). It is imperative to learn more about programs that target this population as well.

Previous review articles examined studies on volunteering, meaningful activities, or social engagement in older adults, but there is less evidence from interventional programs compared to observational studies of self-reported volunteering. For example, Guiney and Machado (7) reviewed 15 studies (4 interventional and 11 observational) on volunteering and cognitive aging up to 2017. Filges et al. (4) examined 24 studies (21 observational and 3 on interventional programs) up to 2019 on the effects of volunteering on health in older adults. In a systematic review dedicated to interventions, Owen et al. (8) identified eight interventions up to 2020 based on various purposeful activities for older adults, of which only two were based on volunteering (9, 10). Together, these reviews focused on the existence and magnitude of associations between volunteering and healthy aging (therefore, only studies that reported quantitative results were included). On the other hand, qualitative evidence has also been reviewed. For example, Tierney et al. (11) reviewed qualitative or mixed-method evidence on adults' (aged older than 18 years, including older adults) perceptions, attitudes, and feelings related to volunteering in various settings up to 2020. These previous reviews shed light on the benefits of volunteering. However, overall, there is a lack of reviews dedicated to volunteering-based interventional programs that specifically recruit older volunteers, and the different types of strategies that are feasible have been less discussed but may inform future innovations. Furthermore, most evidence to date is from the Western world. For example, in a review of formal volunteering programs across the world, 30 out of 54 studies included were from North America or Europe (11). Programs in other parts of the world are less documented.

### The present review

The primary goal of this review is to summarize and evaluate different types of volunteering-based programs involving older adult volunteers. We aim to contribute to the literature in several ways. First, we include programs for older volunteers with and without cognitive impairment. Second, compared to previous reviews (4) focusing on the magnitude of effects (i.e., effect sizes) of volunteering on health outcomes, we aim to present examples of different types of programs that have been feasible. Third, attention is paid to recent evidence, including innovative endeavors sprouting during the COVID-19 pandemic, and efforts are made to include programs from different parts of the world. We review example programs for older volunteers with and without cognitive impairment, respectively, by describing their goals, designs, and intended or actual effects and evaluating the strengths and challenges of the programs and activities.

## Methods

We conducted a non-systematic literature search using Google Scholar and PubMed in November 2022. We used the following search strategy: we first searched studies regardless of the volunteers' cognitive status by using volunteer AND older AND (intervention OR program OR trial OR protocol); then, we specifically searched studies involving cognitively impaired older volunteers by using volunteer AND older AND (intervention OR program OR trial OR protocol) AND (dement OR Alzheimer's OR cognitive impair).

For his review, we searched for peer-reviewed articles published in English and Chinese languages. For relatively recent evidence, we focused on articles published in the year 2010 or after (older studies that provide additional information on the included programs were mentioned where appropriate). With our goal of covering examples of various strategies, qualitative, quantitative, mixed-method studies, and study protocols were considered. Review articles were also searched for any programs or interventions we missed, and if any were located, empirical articles about them were searched. Eligible empirical articles were required to:

1. Document a program or intervention that is implemented through an organization or institution and specifically recruits older adult volunteers (age ≥ 60; the inclusion of younger volunteers as part of the program does not make it ineligible);
2. Describe the program or intervention's goal and design, especially the specific volunteer activities; and 3. report the influences (or, for protocols, intended influence) of the program or intervention on the older volunteers, which may include but are not limited to physical, cognitive, and psychosocial health outcomes.

Of articles/programs that met the inclusion criteria, the final selection of examples was based on several considerations. We tried to include examples from different geographical areas and cultures. Both traditional (in-person) and non-traditional (remote) forms of activities were covered when available. Finally, programs that were not extensively discussed in previous reviews are prioritized.

## Results

**Figure 1:**
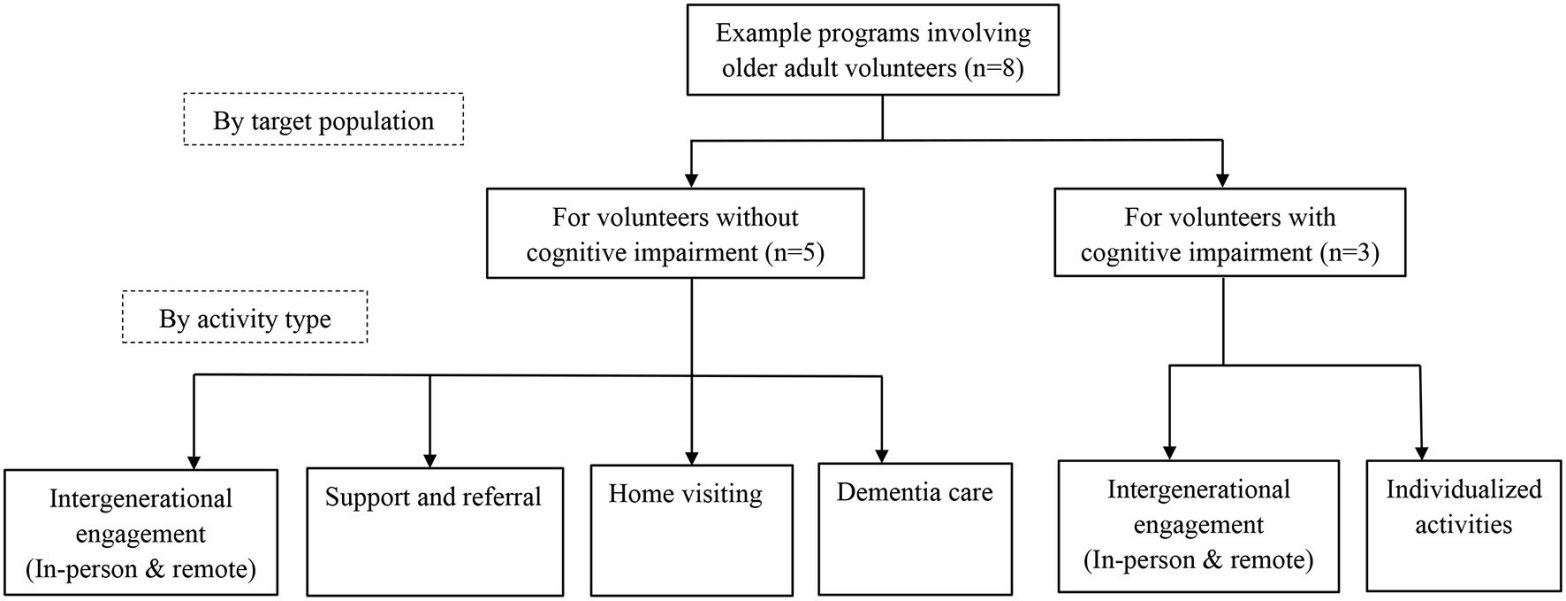
Grouping of included example volunteer programs.

We found articles written in English that met our inclusion criteria but did not find eligible articles in Chinese. Of the English articles that met our inclusion criteria, we identified and selected eight example programs that involve older volunteers with (*n* = 3) and without (*n* = 8) cognitive impairment. Figure 1 shows the grouping of the eight programs, and Table 1 presents a summary of the programs with study findings. These examples cover several representative types of volunteer activities. For older volunteers without cognitive impairment, the activities included intergenerational engagement (both in-person and online forms), support and referral hotline, home visiting, and dementia care services. In the example programs that specifically recruit older volunteers with cognitive impairment, intergenerational engagement (in-person and online forms) and individualized volunteer activities were involved. Below, we summarize the goals, design, and outcomes of each included example program. Of note, although no eligible articles in Chinese were found, the English articles included cover programs and populations from Asia. In addition, some documented volunteer activities that Chinese older adults typically engage in are peer support and help in daily life, community safety, cleaning and maintenance, conflict management, and teenager mentoring (19) (p. 22).

**Table 1:** Summary of included programs.

**Program, Country/Region**	**Volunteer participants**	**Type of activity**	**Studies**	**Main findings**
Research on Productivity through Intergenerational Sympathy (REPRINTS), Japan	Older adults without cognitive impairment	Intergenerational engagement (in-person)	Murayama et al. (12) and Fujiwara et al. (13)	Compared to controls, volunteers showed increased sense of meaningfulness; those who volunteered intensively showed increased self-rated health.
Oasis, US	Older adults without cognitive impairment	Intergenerational engagement (remote)	Sun et al. (14)	Volunteers reported that they used their time productively, increased social activities, and felt better about themselves due to volunteering. They also reported challenges with engaging and developing a relationship with the students remotely and technical issues.
Phone Angel Program, US	Older adults without cognitive impairment	Support and referral	Mui et al. (15)	Volunteers reported feelings of happiness and empowerment, a sense of purpose, and improved communication skills due to the program. They felt that they contributed to the community and improved their social engagement, relationships, and emotional competence.
Age Concern, New Zealand	Older adults without cognitive impairment	Home visiting	Breheny et al. (16)	The home visiting experience led the volunteers to see the challenges associated with aging, recognize the strengths in the people they served, anticipate how they would adapt to their own future aging, and gain a better understanding of life by learning from people with different trajectories of aging.
The Stepping Stones, US	Older adults without cognitive impairment	Dementia care	Han et al. (17)	Volunteers saw their experience as meaningful to themselves as well as the service recipients, enjoyed the teamwork, and learned more about dementia and PwD. Overall, they experienced satisfaction with the interaction and connectedness they had in the program.
The Intergenerational School (TIS), US	Older adults with cognitive impairment	Intergenerational engagement (in-person)	George and Singer (9)	Volunteers had significantly lower levels of stress postintervention compared to baseline.
Seniors Promoting English Acquisition and Knowledge (SPEAK)! Program, US	Older adults with cognitive impairment	Intergenerational engagement (remote)	Piette et al. (18)	Volunteers reported the program being engaging and feeling comfortable working with the language learners (service recipients).
Volunteering-in-Place (VIP), US	Older adults with cognitive impairment	Individualized activities	Klinedinst and Resnick (10)	No significant changes in sedentary activity and psychological health outcomes were observed from baseline to 3–6 months in the program.

### Programs for older volunteers without cognitive impairment

#### Intergenerational engagement

A major type of program involves intergenerational engagement, which often includes teaching, mentoring, and companionship. One exemplar program is the Experience Corps^®^ (EC) in the US, which is an intergenerational tutoring program and model that has been extensively documented (20). We, here, focus on introducing a similar program from the East: The Research on Productivity through Intergenerational Sympathy (REPRINTS) in Japan. In addition, we identified an example of remote volunteering as part of the Oasis, a US non-profit organization.

The primary goal of the REPRINTS (12, 21) was to provide meaningful intergenerational engagement and thereby promoting healthy aging in older adults. In this program, older volunteers (aged ≥ 60 years) visit schools, kindergartens, and childcare settings once per 1–2 weeks to read books to the children and play games with them. Before the formal volunteering phase, older adults receive weekly training on selecting and reading picture books for 3 months. In non-randomized studies (12, 13), REPRINTS volunteers (intervention group) were compared with a control group recruited from various social clubs (but none of the controls engaged in intergenerational programs). It was found that compared to the controls, REPRINTS volunteers showed an increased sense of meaningfulness during 2 years in the program (12), and those who volunteered intensively (for multiple sessions per month) showed increased self-rated health (13).

Volunteering, like many other activities, is challenging during the COVID-19 pandemic. Researchers and practitioners have developed remote forms of strategies. The Oasis' long-standing (since 1989) in-person intergenerational tutoring program transitioned to pen pal letter writing and online tutoring during the pandemic (14). With similar programs, one major goal of the Oasis is to benefit healthier aging through intergenerational engagement. Older volunteers were paired with school-aged children and completed volunteer activities with assistance and guidance from teachers and staff. They engaged in a letter exchange with the children or online reading *via* videoconferencing sessions. Contents of the letters and online conversations included introducing each other and reading and discussing books, following guidelines. In a mixed-method study (14), ~40% of 61 older volunteers (aged 60–79 years) as part of the program reported that they used their time productively and increased social activities, and ~80% of them felt better about themselves due to volunteering. They also reported that engaging and developing a relationship with the students remotely was difficult, technical issues with online tutoring were challenging, and the frequency of exchanging letters was low. With both its challenges and value, remote volunteering was preferred over having no volunteer activities.

#### Support and referral hotline services

One example of the program that involves providing support and referral is the Phone Angel Program in the US (15). In this program, Chinese American older volunteers work with fellow Chinese immigrants who are caring for a sick relative. The goal was to promote social interaction, psychological wellbeing, and skills related to volunteer work (e.g., problem-solving) in the volunteers as they help the service recipients. The program was piloted with 18 volunteers (aged 64–86 years, 72% women) who served 28 caregivers. After being trained by professionals, the volunteers provided advice, referrals, and emotional support for the clients regarding caregiving and family issues via phone calls. Each volunteer worked with one to three caregivers, making one to three calls to each person per week, for up to 6 months. A post-program survey suggested that all volunteers reported feelings of happiness and empowerment, a sense of purpose, and improved communication skills due to the program. Over half of them also felt that they contributed to the community, improved their social engagement and relationships, and the ability to understand their own emotions, among other benefits (15).

#### Home visiting services

Home visiting services, here, refer to structured social services where volunteers visit community-dwelling older adults who tend to be home-bound and socially isolated (16). An example is the home visiting service provided by Age Concern, a charitable organization in New Zealand. The goal of this national service of Age Concern is to alleviate social isolation and feelings of loneliness in community-dwelling older adults (16). Although the primary aim was more to benefit the service recipient, the influeznce on the volunteers has also been examined. Volunteer visitors of the program are accredited and assigned to their service recipients, both typically being older adults. Compared to the service recipients, the volunteers are younger or not housebound if they are older. The volunteers regularly visit the service recipients at their homes, engage in friendly conversations, and provide companionship (16). In a qualitative study, Breheny et al. (16) interviewed six volunteer visitors (aged 68–90 years) and analyzed how the volunteering experience shaped their perceptions of aging. The results suggested that the visiting experience led the volunteers to see the challenges associated with aging (e.g., being treated unfairly in society), recognize the strengths in the people they served, anticipate how they would adapt to their own future aging, and gain a better understanding of life by learning from people with different trajectories of aging.

#### Dementia care services

Another type of setting where older adults volunteer is dementia care (22). The Stepping Stones (17) in the US is an activity-based program for people with dementia (PwD). The main goal of the program was to promote activity engagement in this group of people. For 1.5 h per week, volunteers actively facilitate PwD in participating in appropriate group activities by creating a friendly climate, serving as a reassuring company, and providing practical help with completing an activity. Han et al. (17) analyzed the perspectives of eight older volunteers (aged 67–83 years) on their experience volunteering in the program. The results suggested that the volunteers saw their experience as meaningful to themselves as well as the service recipients, enjoyed the teamwork, and learned more about dementia and PwD. Overall, they experienced socioemotional satisfaction with the interaction and connectedness they had with PwD, the family members of PwD, as well as peer volunteers.

### Programs for older volunteers with cognitive impairment

#### Intergenerational engagement

For programs and interventions that include older volunteers with cognitive impairment, intergenerational engagement remains to be a major type of activity. While various types of intergenerational programs involving this group of people exist (6), we identified The Intergenerational School (TIS) in the US (23) as one example, as its volunteerism component is clear, and its effects have been examined through a randomized study (9).

The goal of TIS is to promote intergenerational interaction between older adults and children. Volunteers with mild-to-moderate dementia were paired with children and engaged in book reading and mentoring. Partnering with the program, George and Singer (9) conducted an intervention study that lasted for 5 months. The objective of the study was to assess the effects of volunteering on the quality of life of older volunteers. They recruited 15 participants living with dementia (mild-to-moderate) from an assisted living facility and randomly assigned them to the intervention (*n* = 8) and control groups (*n* = 7; mean age ~80 years for both groups). The intervention group engaged in volunteering with the children (aged between 5 and 14 years) at TIS. Activities included reading, writing, singing (with kindergarten children), and intergenerational reminiscence (with 6th-grade children). On the other hand, the control group participated in peer seminar sessions on successful aging at their assisted living facility. The hours invested were equal between the groups. The results showed that the intervention group had significantly lower levels of stress postintervention compared to baseline, while stress increased for the control group.

As with programs for cognitively healthy volunteers, remote strategies have also been developed for cognitively impaired older volunteers. One example is the Seniors Promoting English Acquisition and Knowledge (SPEAK)! program in the US developed and piloted by Piette et al. (18). The goal of this program is to promote social engagement and purpose in the life of older adults with mild cognitive impairment (MCI). English-speaking older adults with MCI (*n* = 10, aged 61–89 years) were paired with English language learners (ELL; *n* = 10, aged 18+ years). The participants engaged in language mentoring and social interaction through a videoconferencing platform. They joined a 1-h online conversation session per week for up to 6 weeks. Each pair and their sessions were facilitated by a liaison. In Piette et al. (18) interview with the program participants, the volunteers reported the program being engaging and feeling comfortable working with the ELLs, and no negative experience was reported. Gratitude was expressed by both parties of the pairs.

#### Individualized activities

We identified a US program called Volunteering-in-Place (10) that involves individualized activities for older volunteers with MCI. The goal of VIP is to provide volunteer activities for older adults with MCI who are residents of assisted living facilities. In the program, the older adults engage in volunteer activities that they were interested in and capable of doing (e.g., making and donating food and clothes to the homeless). Volunteers' interests and capabilities were assessed by the program staff to help them find matching activities. Activity fit, enjoyment, and participation were regularly evaluated. Volunteers were allowed to try different activities before they find their favorite one, or they may participate in multiple activities. In a pilot study (10) that examined the preliminary effects of the program, 10 volunteers (average age = 88.12 years) of the program were assessed on sedentary activity and psychological health outcomes, but no significant changes were observed from the baseline to 3 and 6 months in the program.

## Discussion

In this review, we summarized and evaluated programs and intervention studies conducted on the programs that involve various types of volunteer activities for older adult volunteers. We highlight the following lessons learned from these programs.

### Program goals, activities, and outcomes

Of the eight example programs we reviewed, four (REPRINT, Oasis, SPEAK!, and VIP) primarily aimed to benefit the volunteers, two (Phone Angel—with a greater focus on the volunteers and TIS) have the dual goal of benefiting both the volunteers and service recipients, and two (home visiting of Age Concern and Stepping Stones) have the primary aim to benefit the service recipients. Program goals that centered on the volunteers included promoting activity participation, social engagement, wellbeing, and healthy aging overall. The programs covered five major types of activities as follows: intergenerational engagement (mainly including mentoring, pen pal letter writing, and companionship), support and referral hotline, home visiting, dementia care, and individualized activities. Quantitative analyses suggested that volunteering was associated with reduced stress, improvement in self-rated health, psychological wellbeing, and positive feelings about oneself among the volunteers, or no changes in sedentariness and psychological health. Qualitative evidence suggested that perceived benefits (e.g., social satisfaction; gaining practical skills), challenges (technical issues with remote volunteering), and more complex subjective experiences (e.g., a deeper understanding of aging) were reported by volunteers.

### Strengths and challenges

Both strengths and challenges can be identified in volunteering-based programs. First, the strength of this study is the match between the volunteer activity and the volunteer's capability, interest, knowledge, and skills. For example, picture book reading in the REPRINTS (12) was appropriate regarding the level of complexity and difficulty. This activity was both intellectually stimulating and manageable for the older volunteers. Some activities also match volunteers' unique backgrounds and skills in particular domains. For example, in the Phone Angel program (15), having similar experiences and speaking a common language (Chinese) with the service recipients allow the volunteers to provide practical support in the most needed way while helping to alleviate linguistic and cultural isolation. The strength of “match” was probably best demonstrated by the individualized activities in VIP. However, while this model can be crucial for volunteers with cognitive and functional limitations, it might not be feasible and cost-effective in all settings. Generally, finding the match before (e.g., when looking for volunteer opportunities of interest) or during recruitment would be important.

Another strength of the programs is that they serve to meet important psychological needs, including a sense of purpose, self-worth, efficacy, and belongingness. Indeed, meeting psychological needs is one important mechanism through which volunteering benefits health (3). In particular, one way through which these needs are met is the assignment of a social role. In the Phone Angel program, the volunteers are called “Phone Angels” (15), a role that gives them a sense of self-worth and efficacy. In the mentoring-based programs reviewed, volunteers are assigned the role of a mentor, which means they are in a mentor-mentee relationship with the service recipients. This title and role not only indicate their knowledge and experience but also bring an identity and feelings of usefulness. In several studies, participants indicated feeling good about themselves by knowing that they are doing something good for others. Nevertheless, a role that is not clear may bring negative effects as well. In a study (22), volunteers of dementia care programs in Norway reported confusion regarding their roles. It was suggested that volunteers are to supplement but not replace professionals, and their roles need to be clarified to maintain participation (22).

Furthermore, in-person and remote volunteer programs have a unique value. In-person volunteering offers irreplaceable face-to-face interaction, and it is conducive to learning the contexts of each other's lives and building stronger relationships (14). However, in-person participation can be challenging during times such as the COVID-19 pandemic or for individuals with various health-related conditions and limitations. Remote programs offer the possibility of volunteering from home. The value of remote volunteering is recognized and appreciated by volunteers and service recipients (14, 18). Nevertheless, issues raised for remote programs include technical challenges with using online platforms, unsatisfactory speed and frequency in pen pal letter writing, and difficulties in having to engage in mentoring sessions or conversations online (14). Therefore, extra support with technology and the overall management of remote programs are needed to provide a more satisfying experience for older volunteers and service recipients. Together, the development and improvement in both modes of volunteering would help to engage older volunteers of various capabilities and needs.

Regarding older adults with cognitive impairment, our review suggests that of volunteer activities suitable for healthy older adults, some (e.g., intergenerational mentoring) can be appropriate for this population as well, and both in-person and remote modes can be feasible. One way this type of engagement was made possible for volunteers with cognitive impairment was that the programs capitalized the the strengths of these individuals. For example, being fluent in a language that others are learning and becoming language mentors [the SPEAK! Program (18)] helped these older adults recognize not only a personal skill they possess but also a way in which they can contribute to society as well as their peers. In addition, the online mode of the program enables them to volunteer while being in an environment with which they are familiar and comfortable. Most programs recruiting older adult volunteers are designed for those who are relatively healthy cognitively and physically (24). Some barriers to the recruitment and participation of people with cognitive impairment may include functional and cognitive limitations, transportation, safety concerns, the demands and commitment of formal volunteering, and the need for support and help from caregivers (10, 24). Based on our review, there seem several important ways through which the inclusion of older volunteers with cognitive impairment may be promoted. First, the availability of volunteer opportunities should be more widely known to this population and their caregivers. Second, with programs for healthy older adults, finding a good match between people with cognitive impairment and volunteer activity is crucial. Third, alternatives to in-person volunteering, such as online volunteering, may be more manageable and feasible for this population and their caregivers. On a cautious note, questions remain regarding the salience of the prosocial aspect of the activities to this population is not clear. To what extent the effects of volunteer activities are different from other social activities remains to be clarified (25).

### Limitations of this review and notes of caution

We note several limitations of this review. First, a small number of example programs were included based on our inclusion criteria and considerations noted in the Introduction. This selection was not an exhaustive list of all existing programs or activities, and future studies may provide a more encompassing summary, including the most recent innovations. Second, program effects reported in the included studies were mostly based on small samples. The effects of programs with larger samples should be examined in future studies, especially systematic reviews and meta-analyses. Third, among the included studies, there is a need for more rigorous designs with an active control group, such that the effects of volunteering independent of mere social interaction on healthy aging can be elucidated. Furthermore, although we attempted to include programs and studies from different cultures and regions, most examples that could be identified and selected are still from the US and Europe. Future reviews may examine the effects of similar programs in different cultural groups. Finally, we add a note of caution that we do not intend to promote volunteering or active social engagement as normative ideals for aging (26). The fact that challenges, such as health and functional limitations, may prohibit older adults from volunteering should be fully recognized. For older adults who are interested in and capable of volunteering, they may find a program that matches their interests, capabilities, and skills, and more innovative programs may emerge.

## Conclusion

In this mini-review, we summarized different types of volunteering-based programs for older volunteers and evaluated the strengths and challenges of the programs. Volunteer programs, in-person and remote, involving various types of activities, are available for older volunteers with and without cognitive impairment.

Programs strive to match volunteer activities with older volunteers' interests, capabilities, and skills and meet their psychosocial needs. Challenges such as health conditions in older adults and COVID-19-related restrictions have motivated professionals (and older volunteers as well) to be adaptive. Despite heterogeneity in goals, activity type, and outcomes, the programs may shed light on future directions for the development of innovative strategies for healthy aging.

## Author contributions

XZ: conceptualization, methodology, and writing—original draft. ZD, YW, and D-WX: writing—review and editing. All authors contributed to the article and approved the submitted version.
